# Healthcare-seeking behaviors and barriers among medical students in Egypt: a national cross-sectional study

**DOI:** 10.1186/s12889-025-21791-8

**Published:** 2025-02-24

**Authors:** Ghada O. El-Khawaga, Yusof M. Omar, Mohamed Elsaadany, Mohamed Ghazy, Amr Khaled Abdelaal, Mahmoud Hekal, Abdel-Hady El-Gilany, H. S. B. team of collaborators, Huda Wagih, Mey E. Bastawi, Tala Abu Khalaf, Youssef Ahmed, Mazen Essam, Nour Edin Darwish, Mohamed Mahmoud Elfagal, Sondos Hamdy, Abdelrahman Mahmoud Abady

**Affiliations:** 1 Public Health and Community Medicine Department, Faculty of Medicine, Mansoura University, Mansoura, Egypt; 2 Faculty of Medicine, Mansoura National University, Mansoura, Egypt; 3 Faculty of Medicine, Mansoura University, Mansoura, Egypt; 4 Faculty of Medicine, Cairo University, Cairo, Egypt; 5 Faculty of Medicine, Sohag University, Sohag, Egypt; 6 Faculty of Medicine, Fayoum University, Fayoum, Egypt

**Keywords:** Healthcare-seeking behaviors, Barriers to Healthcare, Medical students, Egypt

## Abstract

**Background:**

Medical students are particularly vulnerable to acquiring various health conditions due to the nature of their education but often hesitate to consult doctors and resort to self-managing their illnesses. This study aims to examine the healthcare-seeking behaviors and barriers among medical students in Egypt.

**Methods:**

This cross-sectional study involved a convenience sample of 1,535 medical students from 12 public universities across Egypt. Data on healthcare-seeking behaviors, barriers, and associated factors were collected using an online survey distributed through official university channels and social media platforms. Logistic regression analysis was used to identify factors associated with consulting a doctor when experiencing health problems.

**Results:**

Only 52.2% of participants reported consulting doctors when ill. Participants from rural backgrounds (COR = 0.69, CI: 0.51–0.93), those facing barriers to healthcare (COR = 0.80, CI: 0.66–0.98), with less than sufficient income (COR = 0.61, CI: 0.46–0.81), or a non-working mother (AOR = 0.76, CI: 0.58–0.99) were less likely to consult a doctor. In contrast, participants with more than sufficient income (COR = 1.35, CI: 1.02–1.78), a parent in healthcare (AOR = 13.18–17.31, CI: 6.87–36.35), or who exercised regularly (AOR = 1.48, CI: 1.09–2.01) were more likely to consult a doctor. A substantial proportion of students reported relying on past experiences with the illness (54.1%), using the Internet (46.8%), and self-diagnosing (36.2%). The most common reported barriers to seeking healthcare were lack of time (29.2%), long wait times (22.7%), and academic concerns (12%). A history of feeling uncomfortable or discriminated against when seeking healthcare was reported by 19.5% of participants.

**Conclusion:**

Many medical students in Egypt engage in self-diagnosis and self-management rather than consulting doctors when ill. Addressing healthcare barriers is crucial for encouraging appropriate care-seeking behaviors within this population.

**Supplementary Information:**

The online version contains supplementary material available at 10.1186/s12889-025-21791-8.

## Introduction

Healthcare-seeking behaviors refer to the actions individuals take when they perceive symptoms or conditions requiring evaluation by a healthcare professional [1]. These behaviors are critical determinants of health outcomes, as delays or avoidance in seeking care can lead to complications [2], increased healthcare costs [3], and poorer prognosis [4]. However, the threshold for recognizing and responding to health-related stimuli varies widely among individuals and is shaped by a complex interplay of personal and sociocultural factors, including symptom perception [5], physical and mental health status [6], and external barriers such as structural or financial constraints [7]. Consequently, differences in healthcare-seeking behaviors across populations contribute to disparities in health outcomes, often exacerbating adverse effects in vulnerable groups [8].

Historically, access to health information—and consequently healthcare—was largely restricted to consultations with healthcare professionals. However, the digital age has introduced alternative methods for accessing health-related knowledge, especially among younger populations such as students [9]. University students, in particular, are at the forefront of this shift, utilizing the internet, social media platforms, and telemedicine services to access medical advice, health information, and even remote consultations.

Multiple studies have reported university students using the Internet and social media as their main source of health information [10, 11].This trend raises significant concerns, as health misinformation is incredibly common online, with some studies reporting posts containing misinformation reaching up to 87% [12], and it can have serious consequences on both individual and community levels [13]. Furthermore, this shift in healthcare-seeking behaviors has resulted in increasing reports of self-diagnosis [14], self-medication [15], and self-management among students, particularly medical students—all of which are associated with adverse health outcomes [16].

Medical students are at an increased risk of contracting various illnesses and pathogens during their medical education and training [17]. This is further compounded by the chronic stress they face, which suppresses their immune systems and increases their risk of infection and illness [18]. Despite these risks, studies have shown that medical students often delay seeking medical care, relying instead on family and friends [19].

Recent research on healthcare-seeking behaviors among medical students in Nepal revealed that over 40% of respondents sought help only when their symptoms worsened. [20]. A similar study in Turkey reported that more than half of medical students hesitated to visit the hospital when they were ill [21]. Meanwhile, research conducted among medical students in Egypt has identified several challenges that deter them from seeking help, including cultural stigma, long wait times, and a preference for managing their health problems independently [10]. However, there remains a notable gap in the literature regarding the broader spectrum of healthcare-seeking behaviors among medical students in Egypt, especially regarding the sociocultural and personal factors that influence their decisions.

This study fills a critical gap in the literature by providing a comprehensive analysis of healthcare-seeking behaviors, barriers to timely care, and associated sociocultural influences among medical students in Egypt. Unlike prior research, which has often focused on isolated aspects such as mental health or online health information, this study offers a more holistic examination of the factors that shape healthcare access in this population. Through a national cross-sectional study, we investigate the associations between healthcare-seeking behaviors and various sociodemographic, behavioral, and health-related factors, offering novel insights to inform targeted policies and interventions.

## Methods

### Study design and study period

A cross-sectional study with descriptive and correlational components was conducted between February and October 2024. This study followed the STROBE guidelines for reporting observational studies [22].

### Outcome measures

The primary outcome was defined as consulting a doctor when experiencing health problems [23]. It was measured by the question, "When you experience health problems, who do you usually consult first?".

### Sample size

The sample size was calculated using OpenEpi software [24]. As the prevalence of consulting a doctor when experiencing health problems among medical students in Egypt is definitely unknown, we assumed a 50% prevalence to get the maximum sample size. With a precision of 5%, a confidence level of 95%, and a design effect of 4 to account for the bias introduced by convenience sampling, the calculated sample size was 1,535 students.

### Population

The study population consisted of medical students enrolled in public universities in Egypt, where students have access to healthcare services through student hospitals at minimal or no cost. Medical students from private universities were excluded, as they typically do not have access to such services and rely on private healthcare providers.

### Sampling and data collection approach

A convenience sampling approach was used due to practical constraints, particularly the lack of a feasible method to contact all medical students for simple random sampling. To reduce potential sampling bias, participants were recruited from a diverse range of geographically distributed universities, representing different socioeconomic backgrounds, including universities from Upper Egypt, Lower Egypt, Urban Governorates, and Frontier Governorates. An online survey was administered through Google Forms, ensuring secure and anonymous data collection. Data was collected from medical students enrolled in 12 Egyptian public universities according to the availability of local collaborators. The form was distributed through official channels and groups on Telegram and other social media platforms of the included Egyptian medical schools. Data collection from each medical school was overseen by at least one research assistant, disseminating the questionnaire and making it accessible to students of all academic years. After consenting to participate in the study, participants answered the questionnaire anonymously at their own time.

### Study tools

The self-administered English questionnaire used in the study was developed based on a comprehensive literature review of factors known to influence healthcare-seeking behaviors, as well as questionnaires from previous studies [20, 25–27]. Since the questionnaire did not include scales or constructs requiring psychometric evaluation, formal validity and reliability testing was not conducted. However, to ensure its clarity and relevance, the questionnaire was reviewed by two public health experts and one of the authors, a medical student, who confirmed its appropriateness for the participants. An English version of the questionnaire is provided as a supplementary file (Supplementary File 1).

The first section covered the participants' sociodemographic, behavioral, and health status data. This included variables such as age, sex, academic year, GPA, residence, and living arrangements during the academic year. Family-related factors such as family income, parental education, and parental occupation were also assessed. Additionally, smoking habits, self-reported BMI, physical activity levels, self-perceived general and mental health, the presence of chronic diseases, and health insurance status were recorded.

The second section evaluated the participants' healthcare-seeking behaviors. This included their usual behavior when experiencing illness, such as consulting healthcare professionals, self-diagnosis, self-prescription, or delaying medical care. Their preferred places of care, sources of health information, and the primary reasons influencing these behaviors were also explored.

The third section aimed to identify the prevalence and types of barriers participants faced in accessing healthcare services. It assessed obstacles such as time constraints, financial limitations, lack of knowledge about available services, concerns about academic performance, confidentiality, and social stigma.

### Statistical analysis

In this study, Jamovi Software was used to analyze the collected data. [28] Categorical variables were summarized as frequencies and percentages. The primary dependent variable in this study was whether participants consulted a doctor when experiencing health problems. The independent variables included a comprehensive set of sociodemographic, behavioral, and health-related factors. Variables were implemented in both a univariate and a multivariate binary logistic regression model to assess the independent factors associated with consulting a doctor on experiencing health problems. Only variables with *p*-values less than 0.05 in the univariate regression were included in the multivariate model. Crude and adjusted odds ratios (OR) and their 95% confidence intervals were calculated, and a *p*-value less than 0.05 was considered statistically significant.

Multicollinearity was assessed using Generalized Variance Inflation Factors (GVIFs), and model fit was evaluated using Nagelkerke pseudo R^2^, Akaike Information Criterion (AIC), Bayesian Information Criterion (BIC), and the overall model test. The model explained 36.0% of the variance (Nagelkerke R^2^ = 0.360), with GVIFs below 2, indicating no multicollinearity. AIC and BIC values were 1724 and 1942, respectively, and the overall model test yielded χ^2^ = 460, with a *p*-value < 0.001.

## Results

### Participant demographics

Participants' sociodemographic characteristics are displayed in Table 1. The survey was completed by 1535 students. The sample was evenly distributed across age groups, sex, academic years, and locations, with a median age of 21 and a 1:1.1 female-to-male ratio. Frontier governorates were underrepresented (3.5%). Most participants lived with families (58.4%), in urban areas (74%), with sufficient income (75.3%). About 20% had a parent in a health-related occupation, and most reported good health (Table 2). Additionally, 42.1% reported barriers to healthcare, and 19.5% felt uncomfortable or discriminated against when seeking services (Table 3).

**Table 1:** Distribution of healthcare-seeking behaviors of the medical students in Egypt when feeling ill (*N* = 1535)

Item	n (%)
**First source of consultation**	
Doctors	801 (52.2%)
Family	532 (34.7%)
Peers	152 (9.9%)
Other^a^	50 (3.2%)
**Usual behavior when feeling ill**^b^	
Manage from previous experience	831 (54.1%)
Search the Internet	718 (46.8%)
Self‐diagnose	556 (36.2%)
Ignore problem	498 (32.4%)
Consult a doctor in a non‐medical setting	448 (29.2%)
Self‐prescription	428 (27.9%)
Consult pharmacist	363 (23.6%)
Seek immediate care	264 (17.2%)
Ask peers to perform examination	183 (11.9%)
Regular check‐ups	165 (10.7%)
**Usual place of care**	
Private	1141 (74.3%)
University hospital	202 (13.2%)
Student hospital	99 (6.4%)
Faculty clinic	73 (4.8%)
Home	20 (1.3%)
**Primary consideration when seeking healthcare**	
Quality of care	887 (57.8%)
Doctor's reputation	305 (19.9%)
Accessibility	220 (14.3%)
Cost	123 (8.0%)

^a^Other includes pharmacist, self, and friends, ^b^Categories are not mutually exclusive

**Table 2:** Sociodemographic characteristics of medical students associated with consulting a doctor when experiencing health problems (*N* = 1535)

**Variables**		**Total n (%**)^a^	**Doctor consultation n (%)**^b^	**Univariate regression**	
				**COR (95% CI)**^c^	***p*-value**
Overall		1535	801 (51.2%)		
Age	Less than 21	807 (52.6%)	418 (51.8%)	1	
	21 or above	728 (47.4%)	383 (52.6%)	1.03 (0.85–1.26)	0.75
Sex	Female	813 (53%)	410 (50.4%)	1	
	Male	722 (47%)	391 (54.2%)	1.16 (0.95–1.420	0.15
Academic year	1st year	265 (17.3%)	151 (57%)	1	
	2nd year	350 (22.8%)	172 (49.1%)	0.73 (0.53–1.01)	0.05
	3rd year	371 (24.2%)	188 (50.7%)	0.78 (0.57–1.07)	0.12
	4th year	353 (23%)	192 (54.4%)	0.9 (0.65–1.24)	0.52
	5th year	196 (12.7%)	98 (50%)	0.76 (0.52–1.09)	0.14
GPA	Excellent	897 (58.4%)	465 (51.8%)	1	
	Very Good	386 (25.1%)	206 (53.4%)	1.06 (0.84–1.35)	0.94
	Good or less	252 (16.3%)	130 (51.6%)	0.99 (0.75–1.31)	0.62
Residence	Urban	1136 (74%)	629 (55.4%)	1	
	Rural	399 (26%)	181 (45.4%)	0.69 (0.55–0.87)	**0.002**
Geographical location	Urban Governorates	562 (36.6%)	307 (54.6%)	1	
	Lower Egypt	471 (30.7%)	241 (51.2%)	0.87 (0.68–1.11)	0.27
	Upper Egypt	448 (29.2%)	227 (50.7%)	0.85 (0.67–1.09)	0.21
	Frontier Governorates	54 (3.5%)	26 (48.1%)	0.77 (0.44–1.35)	0.36
Living situation	With family	897 (58.4%)	474 (52.8%)	1	
	External residence	460 (30.0%)	246 (53.5%)	0.73 (0.51–1.03)	0.07
	University dormitory	178 (11.6%)	81 (45.5%)	0.98 (0.78–1.22)	0.08
Family income	Sufficient	1156 (75.3%)	597 (51.6%)	1	
	More than sufficient	278 (18.1%)	164 (59%)	1.35 (1.03–1.76)	**0.03**
	Less than sufficient	101 (6.6%)	40 (39.6%)	0.61 (0.41–0.93)	**0.02**
Father education	Higher education	1255 (81.8%)	690 (55%)	1	
	Secondary education	208 (13.5%)	87 (41.8%)	0.41 (0.25–0.68)	** < 0.001**
	Illiterate or primary	72 (4.7%)	24 (33.3%)	0.59 (0.44–0.79)	** < 0.001**
Father occupation	Non-health related occupation	1142 (74.4%)	472 (41.3%)	1	
	Health related occupation	310 (20.2%)	293 (94.5%)	24.47 (14.80–40.46)	** < 0.001**
	Non-working	83 (5.4%)	36 (43.4%)	1.09 (0.69–1.71)	0.72
Mother education	Higher education	1177 (76.7%)	642 (54.5%)	1	
	Secondary education	258 (16.8%)	119 (46.1%)	0.71 (0.54–0.94)	**0.01**
	Illiterate or primary	100 (6.5%)	40 (40%)	0.56 (0.37–0.84)	**0.006**
Mother occupation	Non-health related occupation	636 (41.4%)	290 (45.6%)	1	
	Health related occupation	240 (15.6%)	233 (97.1%)	39.7 (18.4–85.6)	** < 0.001**
	Non-working	659 (43%)	278 (42.2%)	0.87 (0.7–1.08)	0.22

^a^Percentages are column-wise, ^b^Percentages are row-wise, ^c^*COR* Crude Odds Ratio, *CI* Confidence Interval

**Table 3:** Health status and healthcare-seeking patterns of medical students associated with consulting a doctor when experiencing health problems (*N* = 1535)

**Variables**		**Total n (%**)^a^	**Doctor consultation n (%)**^b^	**Univariate regression**	
				**COR (95% CI)**^c^	***p*-value**
Smoking	No	1470 (95.8%)	750 (51.9%)	1	
	Yes	65 (4.2%)	41 (63%)	1.6 (0.96–2.67)	0.08
Weight	Normal	913 (59.5%)	460 (50.4%)	1	
	Overweight	451 (29.4%)	252 (55.9%)	1.25 (0.99–1.56)	0.06
	Underweight	171 (11.1%)	89 (52%)	1.07 (0.77–1.48)	0.69
Chronic diseases	No	1389 (90.5%)	724 (52.1%)	1	
	Yes	146 (9.5%)	77 (52.7%)	1.03 (0.73–1.44)	0.89
Self-perceived general health	Average	582 (37.9%)	288 (49.5%)	1	
	Above average	827 (53.9%)	449 (54.3%)	1.21 (0.98–1.50)	0.08
	Below average	126 (8.2%)	64 (51.6%)	1.05 (0.72–1.55)	0.79
Self-perceived mental health	Average	445 (29.0%)	236 (53%)	1	
	Above average	837 (54.5%)	440 (52.6%)	0.98 (0.78–1.24)	0.87
	Below average	253 (16.5%)	125 (49.4%)	0.86 (0.64–1.18)	0.36
Self-diagnosis confidence	Somewhat confident	990 (64.5%)	520 (52.5%)	1	
	Not confident	464 (30.2%)	235 (50.4%)	0.93 (0.74–1.16)	0.5
	Very confident	81 (5.3%)	46 (56.8%)	1.18 (0.75–1.88)	0.46
Weekly physical activity	Less than 30 min	692 (45.1%)	333 (48.1%)	1	
	More than 30 min	843 (54.9%)	468 (55.5%)	1.35 (1.10–1.65)	**0.004**
Part-time job	No	1405 (91.5%)	734 (52.2%)	1	
	Yes	130 (8.5%)	67 (51.5%)	0.97 (0.68–1.39)	0.88
Health insurance	No	1107 (72.1%)	561 (50.7%)	1	
	Yes	428 (27.9%)	240 (56.1%)	1.24 (0.99–1.55)	0.06
Perceived barriers seeking healthcare	No	889 (57.9%)	485 (54.6%)	1	
	Yes	646 (42.1%)	316 (48.9%)	0.80 (0.65–0.98)	**0.03**
Previous unpleasant experience^d^	No	1235 (80.5%)	653 (52.9%)	1	
	Yes	300 (19.5%)	148 (49.3%)	0.87 (0.67–1.12)	0.27

^a^Percentages are column-wise, ^b^Percentages are row-wise, ^c^*COR* Crude Odds Ratio, *CI* Confidence Interval, ^d^Unpleasant experience was defined as feeling uncomfortable or discriminated against when seeking healthcare services

### Healthcare-seeking behaviors and predictors

Healthcare-seeking behaviors are shown in Table 1. Over half (52.2%) of the participants reported consulting doctors when experiencing health problems. However, self-management strategies were prevalent, with 54.1% of participants relying on past experiences, 46.8% using the internet, and 36.2% engaging in self-diagnosis. Private clinics were the most common place of care (74.3%), with quality of care (57.8%) and doctor reputation (19.9%) cited as key factors when seeking healthcare.

As shown in Table 2, univariate regression identified significant sociodemographic factors associated with consulting doctors when experiencing health problems. Individuals residing in rural areas consulted doctors less frequently than those in urban areas (OR = 0.69, 95% CI: 0.51–0.93). Family income was also a significant factor, with those reporting more than sufficient family income more likely to consult doctors (OR = 1.35, 95% CI: 1.02–1.78), while those with less than sufficient family income were less likely to consult doctors (OR = 0.61, 95% CI: 0.46–0.81). Lower maternal and paternal education levels were associated with less frequent consultations, and having a father or mother with a health-related occupation was strongly associated with more frequent consultations (OR = 24.47, 95% CI: 12.35–48.52; OR = 39.7, 95% CI: 18.64–84.59, respectively). Additionally, exercising more than 30 min per week (OR = 1.35, 95% CI: 1.02–1.78) and facing barriers when seeking healthcare (OR = 0.8, 95% CI: 0.66–0.98) were also significantly associated with consulting doctors.

On multivariate regression, significant factors included having a father (AOR = 13.18, 95% CI: 6.87–25.29) or mother (AOR = 17.31, 95% CI: 8.24–36.35) in healthcare, with those in healthcare more likely to consult doctors. Having a non-working mother was associated with a lower likelihood of consulting doctors (AOR = 0.76, 95% CI: 0.58–0.99), while exercising more than 30 min per week increased the likelihood (AOR = 1.48, 95% CI: 1.09–2.01) (Table 4).

**Table 4:** Results of multivariate binary logistic regression for significant factors associated with medical students consulting a doctor when experiencing health problems

**Variables**			**Multivariate regression**	
		**β**	**AOR (95% CI)**^a,b^	***p*-value**
Residence	Urban		1	
	Rural	0.08	0.90 (0.69–1.18)	0.45
Family income	Sufficient		1	
	More than sufficient	-0.21	0.87 (0.63–1.21)	0.41
	Less than sufficient	-0.12	0.78 (0.48–1.27)	0.32
Father occupation	Non-health related occupation		1	
	Health related occupation	2.58	13.18 (7.79–22.3)	** < 0.001**
	Non-working	0.13	1.09 (0.65–1.82)	0.76
Mother occupation	Non-health related occupation		1	
	Health related occupation	2.851	17.31 (7.8–38.3)	** < 0.001**
	Non-working	-0.281	0.76 (0.58–0.98)	**0.037**
Weekly physical activity	Less than 30 min		1	
	More than 30 min	0.392	1.48 (1.17–1.87)	**0.001**
Perceived barriers seeking healthcare	No		1	
	Yes	-0.21	0.82 (0.64–1.03)	0.09

^a^*AOR* Adjusted Odds Ratio, *CI* Confidence Interval, ^b^% Correctly Predicted = 36.0, GIVF < 2, AIC = 1724, BIC = 1942, Model χ^2^ = 460, *p* < 0.001

### Sources of health information and healthcare-seeking barriers

Doctors were the most commonly reported source of health information by the participants (77.9%), followed by the internet (56.6%), and family members (37.5%) (Table 5). The most common barriers to seeking healthcare included lack of time (29.2%), long wait times (22.7%), and concerns about academic performance (12%), unwanted interventions (11.7%), and side effects (11.1%) (Table 6).

**Table 5:** Sources typically utilized by medical students in Egypt for health information (*N* = 1535)

Sources^a^	n (%)
Doctors	1196 (77.9%)
The Internet	869 (56.6%)
Family members	575 (37.5%)
Textbooks	565 (36.8%)
Social media	509 (33.2%)
Curriculum	485 (31.6%)
Peers	440 (28.7%)
Research paper(s)	439 (28.6%)

^a^Categories are not mutually exclusive

**Table 6:** Barriers reported by medical students in Egypt when seeking healthcare (*N* = 1535)

Barrier/reason^a^	n (%)
Lack of time	448 (29.2%)
Long wait times	349 (22.7%)
Unsure where to seek help	264 (17.2%)
Economic cost	215 (14%)
Lack of access to services	188 (12.2%)
Fear of impact on academic performance	184 (12%)
Fear of unwanted interventions	179 (11.7%)
Fear of side effects	170 (11.1%)
Lack of confidentiality/privacy	122 (7.9%)
Lack of transportation	97 (6.3%)
Social stigma	65 (4.2%)

^a^Categories are not mutually exclusive

## Discussion

Medical students are often considered a vulnerable population, with studies suggesting they report higher rates of physical and mental illnesses compared to their non-medical peers [29]. While this pattern is well-documented in other countries, local data in Egypt remains limited. Despite these health concerns, many medical students delay seeking healthcare due to various influencing factors and barriers. Understanding these factors is essential for addressing the issue and developing effective solutions.

In our study, despite better access to healthcare in general and doctors in particular, only about half (52.2%) of students consulted doctors when being ill. These results are similar to studies conducted among health profession students in Uganda [25] and medical students in Hungary [23] and Nepal [20], where 57.5%, 56.8%, and 64% of the participants respectively consulted a physician or health worker as their first action when ill. However, our result is higher than that of another study among medical students in Uganda [26], in which only 20% of the participants consulted doctors when feeling ill. Our results also differ from those of a study among university students in Lebanon [30], out of whom only 35.7% sought health care from a physician or health facility when feeling ill. These differences may be due to differences in healthcare access, cultural norms and attitudes, and perceived severity of illness [31].

In our study, although insignificant on multivariate analysis, participants from rural backgrounds and those with insufficient income were significantly less likely to consult a doctor when feeling ill compared to their more well-off peers. Similar results were reported in a community-based study in Egypt [31], which found that family income was a key determinant of healthcare-seeking behavior, with cost being the primary reason for seeking non-qualified healthcare practitioners. Our results are also in line with that of a study among heads of households in southwest Ethiopia [32], where urban residence and adequate income were associated with better healthcare-seeking behaviors. However, our results differ from that of a study among medical students in Hungary [23], where high income was not significantly associated with seeking healthcare when feeling ill.

The observed differences in doctor consultation behavior based on socioeconomic status in our study likely stem from barriers students face and their healthcare preferences. Most participants identified quality of care as the most important factor when seeking healthcare. However, students with limited income may struggle to afford high-quality healthcare services, while those living in remote rural areas face the challenge of traveling long distances to access reputable providers. This finding aligns with a national survey in Egypt, which highlighted socioeconomic status as significantly associated with timely care-seeking, rather than a preference for private healthcare [33].

Similarly, another Egyptian study among medical students reported that 14.9% of participants identified distance as a primary barrier to seeking healthcare [11]. Addressing these challenges requires targeted policy interventions, such as subsidized healthcare services for students from low-income backgrounds and the expansion of telemedicine services to mitigate the impact of geographic barriers. These measures could help ensure equitable access to healthcare for students, regardless of their socioeconomic status or location.

The increased likelihood of consulting a doctor when feeling ill among participants with parents who work in healthcare in our study may be because growing up, parents are often the first person children turn to when feeling ill [34], especially if the parent also happens to be a healthcare provider or doctor. In concordance, in a study conducted among medical students in Cairo, 50% of the participants reported seeking health information from their parents in the past six months [10]. Similarly, in a study conducted among Indian adolescents [35], parents were reported as the first source of health information by the majority (93%) of the participants.

Our finding of physical activity being significantly positively associated with consulting a doctor when feeling experiencing health problems is in line with a study among medical students in Nepal that found that students who exercised daily were significantly less likely to neglect their well-being [20]. This may be because weekly physical activity and exercise is a reflection of a higher level of health consciousness [36]. Thus, students who allocate the time to be physically active on a weekly basis in their already busy schedules may also allocate more time to taking care of their health in general and would be more likely to consult a doctor when feeling ill.

A significant proportion of medical students in our study reported consulting doctors when experiencing health problems. However, when it came to managing their health problems, a notable tendency toward self-management emerged. This is consistent with other studies among medical students with various health conditions such as acne [37], dysmenorrhea [38], and headaches and migraines [39], where only a minority of the participants reported consulting a doctor for their illnesses and reported self-managing their illnesses themselves. Furthermore, a recent systematic review of self-medication practices reported a self-medication prevalence among medical students twice as high as that of their non-medical peers [40]. Nonetheless, our results are much lower than those of a study among medical students in the United Arab Emirates [41], in which 95.8% reported self-prescribing, and 87.4% reported ignoring the illness entirely.

The high prevalence of self-medication and self-management in general among medical students is often attributed to their increased knowledge of different diseases or medications and their confidence in self-diagnosing and self-treating their illnesses [42]; however, these behaviors carry significant risks, including misdiagnosis, inappropriate treatment, delayed care, and the potential worsening of health conditions [40]. This behavior can be reinforced by the pressures of medical school, where demanding schedules and the high expectations placed on students to perform academically may lead them to overlook their health in favor of their studies [43]. Furthermore, external factors such as perceived system-based barriers, including stigma, confidentiality concerns, and a lack of awareness about healthcare service availability, also contribute to medical students opting for self-management [27, 44]. However, our study revealed that many participants reported poor confidence in self-diagnosing, suggesting that these factors alone cannot fully explain why Egyptian medical students tend to avoid seeking proper healthcare.

One possible explanation is that medical students in Egypt may prioritize their studies and exams over their health, as suggested by 32.4% of participants in our study who reported typically ignoring their illnesses when feeling unwell. This finding is similar to a study conducted among medical students in Cairo, where only 39% of participants reported adequately caring for their health [43]. Another reason could be the dissatisfaction with healthcare in Egypt, which was similarly reported by 59.7% of the medical students in a study conducted in Tanta [11]. This dissatisfaction may drive students to seek private healthcare or delay consulting a doctor, opting to manage their health issues independently [44].

The high reliance on self-management may be further exacerbated by systemic issues within the public healthcare system. University hospitals and student clinics are often underutilized due to long waiting times and concerns about the quality of care, pushing students to seek private healthcare instead [45, 46]. In Egypt, these facilities are frequently staffed by residents who report being understaffed, underpaid, and undervalued [47]. Moreover, healthcare professionals often use these facilities as training grounds with minimal oversight, while many supplement their income through private practice [48]. This trend mirrors findings from Northern Cyprus [49], where 39.1% of students preferred private care, reflecting a broader trust deficit in public healthcare systems. The lack of time and dissatisfaction with the quality of public healthcare, consistently cited as barriers by medical students [11], further drive their preference for private care.

Addressing these challenges requires a dual focus on medical education and healthcare policy reform. Medical education policies should prioritize the establishment of dedicated, high-quality health services for students, with an emphasis on mental health support and health promotion integrated into the curriculum. Concurrently, healthcare policy should focus on enhancing the accessibility, efficiency, and quality of student healthcare services. Streamlining administrative processes, upgrading physical infrastructure, and incorporating telemedicine options can make student clinics more effective and appealing. Additionally, initiatives such as guided tours of these facilities at the beginning of the academic year could build trust and encourage greater utilization of public healthcare resources by students.

### Strengths and limitations

Our study had both strengths and weaknesses. To the best of our knowledge, this is the first study in Egypt to assess healthcare-seeking behaviors, barriers, and associated factors of medical students nationally. A strength of this study is the inclusion of a large sample of students from different academic years across public universities in Egypt’s four regions, though the use of convenience sampling may limit the representativeness of the sample. Additionally, the study’s focus on a resource-constrained setting provides valuable insights that could be applicable in similar contexts globally.

Nonetheless, several limitations should be considered. Convenience sampling may have introduced selection bias, limiting the generalizability of the results. Future studies could use probability sampling for more representative data. The reliance on self-reported data introduces potential recall and social desirability biases, while the use of an English-language questionnaire may have led to some misinterpretation. Additionally, the lack of formal validity and reliability testing of the questionnaire may introduce measurement error. The small sample size from the frontier region and the exclusion of private medical schools further limit generalizability. Future research should include a broader sample to provide a more comprehensive picture.

Despite these limitations, the study makes a significant contribution to understanding the healthcare-seeking behaviors of medical students in Egypt. It offers a foundation for developing targeted interventions, as well as a reference for other low-resource settings aiming to address similar challenges.

## Conclusions

This study highlights critical gaps in healthcare-seeking behaviors among Egyptian medical students, emphasizing the urgency of targeted interventions. Policies should prioritize improving the efficiency of university hospitals, addressing time-related barriers, and fostering a culture of proactive healthcare-seeking through flexible clinic hours and tailored health education. Future research should identify effective strategies to reduce self-management and delayed care. These findings provide actionable insights for developing interventions that not only enhance medical students' health outcomes but also serve as a model for similar low-resource settings.

## Supplementary Information

Supplementary Material 1

## Data Availability

The data of this study is available from the corresponding author upon reasonable request.

## Abbreviations

AIC: Akaike Information Criterion
AOR: Adjusted Odds Ratio
BMI: Body Mass Index
BIC: Bayesian Information Criterion
CI: Confidence Interval
GPA: Grade Point Average
GVIF: Generalized Variance Inflation Factor
Nagelkerke R^2^: Nagelkerke’s pseudo R^2^
OR: Odds Ratio
STROBE: Strengthening the Reporting of Observational Studies in Epidemiology
