# Supplementary material for: Healthcare-seeking behaviors and barriers among medical students in Egypt: a national cross-sectional study
Source: BMC Public Health. 2025 Feb 24;25:743. doi: 10.1186/s12889-025-21791-8 (PMC11849205; doi:10.1186/s12889-025-21791-8)
Supplement: Supplementary file 1 — Supplementary Material 1 [file 12889_2025_21791_MOESM1_ESM.docx]

# Healthcare-Seeking Behaviors and Barriers Questionnaire

## Section 1: Sociodemographic, Behavioral, and Health Status

**1. Age (in years): ________**

**2. Sex:
 - 1. Male
 - 2. Female**

**3. Academic Year:
 - 1. 1st year
 - 2. 2nd year
 - 3. 3rd year
 - 4. 4th year
 - 5. 5th year**

**4. GPA:
 - 1. Excellent (A)
 - 2. Very Good (B)
 - 3. Good (C)
 - 4. Acceptable (D)
 - 5. Failed (F)**

**5. Residence:
 - 1. Urban (City)
 - 2. Rural (Countryside)**

**6. Living Situation During Academic Year:
 - 1. With family
 - 2. University dormitory
 - 3. External residence**

**7. Medical School: (Specify university name)**

**8. Part-Time Job:
 - 1. Yes
 - 2. No**

**9. Family Income:
 - 1. Less than sufficient
 - 2. Sufficient
 - 3. More than sufficient**

**10. Father’s Education:
 - 1. Illiterate or Primary
 - 2. Secondary Education
 - 3. Higher Education**

**11. Father’s Occupation:
 - 1. Non-working
 - 2. Health-related Occupation
 - 3. Non-Health-related Occupation**

**12. Mother’s Education:
 - 1. Illiterate or Primary
 - 2. Secondary Education
 - 3. Higher Education**

**13. Mother’s Occupation:
 - 1. Housewife
 - 2. Health-related Occupation
 - 3. Non-Health-related Occupation**

**14. Smoking:
 - 1. Yes
 - 2. No**

**15. On a typical week, how much time do you spend on moderate and vigorous physical activities?
 - 1. Less than 30 minutes
 - 2. 90-150 minutes**

**- 3. 150-300 minutes
 - 4. More than 300 minutes**

**16. Which of the following best describes your weight?
 - 1. Underweight
 - 2. About right
 - 3. Overweight
 - 4. Obese**

**17. Do you suffer from any chronic diseases?
 - 1. Yes
 - 2. No**

**18. Do you have health insurance?
 - 1. Yes
 - 2. No**

**19. How would you perceive your general health?
 - 1. Average
 - 2. Above Average
 - 3. Below Average**

**20. How would you perceive your mental health?
 - 1. Average
 - 2. Above Average
 - 3. Below Average**

**21. How confident are you in your ability to self-diagnose health problems?
 - 1. Not confident at all
 - 2. Somewhat confident
 - 3. Very confident**

## Section 2: Healthcare-Seeking Behaviors

**1. When you experience health problems, who do you usually consult first?
 - 1. Doctors (including family members who are doctors)
 - 2. Peers (fellow medical students)
 - 3. Family**

**- 4. Others (please specify)**

**2. If you feel sick, how would you describe your usual behavior? (Choose all that apply):
 - 1. Self-prescription
 - 2. Ignore the problem
 - 3. Seek immediate care
 - 4. Self-diagnose
 - 5. Use the internet**

**3. What is the usual place of your healthcare?
 - 1. Private clinics
 - 2. University hospital
 - 3. Student hospital
 - 4. Faculty clinic
 - 5. Home**

**4. Which factor is most important to you when making decisions about your healthcare?
 - 1. Cost
 - 2. Quality of care
 - 3. Accessibility
 - 4. Doctor's reputation**

**5. Where do you typically get your health information? (Choose all that apply):
 - 1. Peers
 - 2. Family members
 - 3. Social media
 - 4. The Internet
 - 5. Curriculum
 - 6. Textbooks
 - 7. Research paper(s)
 - 8. Doctors**

## Section 3: Barriers to Accessing Healthcare

**1. Do you face any barriers accessing healthcare services?
 - 1. Yes
 - 2. No**

**2. If yes, what barriers do you face when seeking healthcare? (Choose all that apply):
 -1. Lack of time
 -2. Long wait times
 -3. Unsure where to seek help
 -4. Economic cost
 -5. Lack of access to services
 -6. Fear of impact on academic performance
 -7. Fear of unwanted interventions
 -8. Fear of side effects
 -9. Lack of confidentiality/privacy
 -10. Lack of transportation
 -11. Social stigma**

**3. Have you ever felt uncomfortable or discriminated against when seeking healthcare services?
 - 1. Yes
 - 2. No**
